# Screening by Self-Report Underestimates Sickle Cell Trait in High-School Athletes

**DOI:** 10.7759/cureus.19247

**Published:** 2021-11-04

**Authors:** Gary Allen, Michael S Smith, Michelle Bruner, Kelli Agrawal, James R Clugston, Bryan R Prine

**Affiliations:** 1 Sports Medicine, Ascension Health, Pensacola, USA; 2 Sports Medicine, University of Florida College of Medicine, Gainesville, USA; 3 Public Health, University of South Florida College of Public Health, Tampa, USA; 4 Community and Family Medicine, University of Florida College of Medicine, Gainesville, USA; 5 Sports Medicine, University of Florida Health, Gainesville, USA

**Keywords:** athlete, physical, pre-participation, screening, sickle

## Abstract

Background: Sickle cell trait (SCT) has received attention as a cause of death in college athletes, leading to mandatory lab SCT screening in National Collegiate Athletic Association (NCAA) athletes. High-school athletes are commonly screened by self-report. There are no known studies for evaluating whether this method is effective as a screening tool.

Hypothesis: The local prevalence rate of SCT as self-reported on the preparticipation evaluation (PPE) forms would be lower than the national accepted average.

Methods: PPE forms from the Department of Orthopedics and Rehabilitation of the University of Florida (UF) were reviewed between January 1, 2017, and April 30, 2018. The Florida High School PPE form includes a yes/no question to assess the diagnosis of SCT. The prevalence established by self-report was then compared with the national prevalence for SCT in the comparable race/ethnicity groups reported by the CDC. The response rate of SCT questions was also compared to other common cardiac screening questions.

Results: A total of 401 forms were reviewed. Six (1.5%) students answered “yes,” 351 answered “no,” and 44 left the SCT question blank. All six athletes who self-reported “yes” were Black and made up 3.7% of the 162 known Black respondents. This self-report rate for Black/African Americans was well below the expected 7.3% described by the CDC. Response rates were also lower than the comparable cardiac screening questions.

Conclusions: Self-report SCT status rates are lower than the accepted prevalence in a similar population. Significant inconsistencies in reporting were also determined.

Clinical relevance: This is a rare study to evaluate the self-reported prevalence of SCT in high-school athletes. Below average reporting of SCT and inconsistency in completion of the forms increase the concern for accuracy and effectiveness of current high-school SCT screening methods relying on self-report.

## Introduction

Sickle cell trait (SCT) is a condition resulting from the inheritance of one gene for sickle hemoglobin (S) and one gene for normal hemoglobin (A). Although predominantly a silent condition, SCT in athletes can pose a range of clinical problems related to the intensity of training. The vital concern is exertional collapse associated with sickle cell trait (ECAST), which can be fatal, can occur in a variety of sports, and was the leading cause of death in National Collegiate Athletic Association (NCAA) Division 1 American College football players in the decade prior to 2010 [1,2]. The proposed mechanism behind ECAST is thought to be related to high-intensity exercises leading to significant erythrocyte sickling, inflammation, and microvascular occlusion with subsequent infarction. The risk of ECAST is heightened with dehydration and thermal strain. The proposed mechanism of death is explosive rhabdomyolysis leading to arrhythmia and/or multi-organ failure [3].

The Centers for Disease Control and Prevention (CDC) report that the incidence of SCT in the general United States population is 1.5%, with 2.5% in the state of Florida, and 7.3% (about one in 13) in Black Americans as a whole. Incidence for White and Hispanic Americans is 0.3% and 0.7%, respectively [4]. SCT has been associated with exertional deaths in NCAA Division I football players with a rate of 1:827, which is 37 times higher than that in athletes without SCT [5]. In addition to sudden death, additional significant health outcomes secondary to exertional sickling include splenic infarction, renal failure, and acute lumbar paraspinal myonecrosis [2,6].

Due to the significant attention SCT received as a cause of death in American football athletes, mandatory screening in the NCAA was initiated for Division I athletes in 2010. Exertional SCT-related deaths were significantly reduced from 48% of non-traumatic sudden death events to only 7% in the years that followed [7]. The screening was subsequently expanded to all NCAA Division II athletes in 2012 and Division III athletes in 2013. Current strategies to prevent death in athletes with SCT include various screening methods, followed by education and targeted interventions focused on modifications of training intensity, hydration, acclimatization, increased surveillance in high-risk environments (heat and high altitude), and cessation of activity with any symptoms [3].

Screening for SCT in athletes at the time of preparticipation evaluation (PPE) continues to be a debated topic due to potential ethical and legal considerations. However, all newborns in the United States (US) are screened for sickle cell disease at the time of birth, thus discovering their SCT status. In the state of Florida, screening for SCT was instituted in 1989 [8]. There is currently a database of SCT status dating back to 2006 with results available upon request by the individual or healthcare provider. However, this information may not be communicated to or recalled by the athletes or their parents at the time of PPE. For collegiate athletes with unknown SCT status, the NCAA recommends a hemoglobin solubility test at a minimum. The athlete has the ability to opt out of the screening process without any risk of jeopardizing their ability to participate in the sport of their choice.

Contrary to the NCAA, the American Society of Hematology (ASH) does not support testing or disclosure of SCT status as a prerequisite for participation in athletic activities, citing potential harm to the student-athlete in the form of stigmatization and racial discrimination [9]. They recommend the implementation of universal interventions instead, which have been described as successfully utilized by the military [10].

High-school athletes in Florida are currently screened based upon the Florida High School Athletic Association’s (FHSAA) PPE EL2 form medical history questionnaire, which asks if the athlete has ever been diagnosed with sickle cell anemia or SCT along with the American Heart Association (AHA) cardiac screening questions [11]. There have been no known studies to date that validate this method of screening for SCT in high-school athletes. The purpose of this study was to attempt to validate this process through the following aims.

Hypothesis

It was hypothesized that the local prevalence rate of SCT as reported on the PPE forms would be lower than the national accepted average.

Study design

Retrospective Clinical Study

The aims of this study are to (1) determine the rate of completion of the two questions pertaining to sickle cell anemia and sickle cell trait on the FHSAA PPE forms and (2) compare the local reported SCT prevalence to the national prevalence described by the Centers for Disease Control and Prevention (CDC).

## Materials and methods

**Figure 1:**
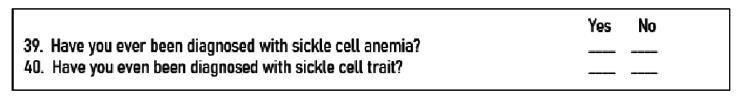
Questions 39 and 40

Under an approved HIPAA (Health Insurance Portability and Accountability Act) full waiver of consent, FHSAA PPE forms were reviewed of middle school- and high-school-aged student-athletes who completed exams at the Orthopedic Sports Medicine Institute (OSMI) clinic between January 1, 2017, and April 30, 2018, and those whose PPE forms were on file. Responses to two questions on the PPE form regarding sickle cell anemia and SCT (questions 39 and 40, see Figure 1) were reviewed and documented. As the race was not included on the PPE form, a medical record chart review was conducted to verify their race. Data were de-identified upon verification of race through the chart review. The rate of completion, the total prevalence of SCT, and the prevalence of SCT by race were calculated and compared with the CDC prevalence rates.

## Results

A total of 401 records were reviewed. Table 1 summarizes the breakdown by race, age, and gender. The highest population groups were males (82%) and Black race (40%), with an average age of 14.7 + 2.2 years. As not all participants were previous University of Florida (UF) Health patients, we were unable to obtain race on 97 records through medical record review.

**Table 1:** Demographics

	Mean Age (years)	Male	Female	Total
Asian	15	6	1	7
Biracial	16	2	0	2
Black	14.4	137	25	162
Hispanic	14.6	4	1	5
Other	14.6	11	2	13
Unknown	15	87	10	97
White	14.8	84	31	115
Total	14.7 + 2.2	33	70	401

Most athletes (n = 396) responded “no” to Question 39, which asked whether they have sickle cell anemia, and five athletes did not answer (three Black and two unknown races). There were 162 confirmed Black athletes of which six (3.7%) responded “yes” and 21 (13%) left Question 40 regarding the diagnosis of SCT blank (unanswered). This was 50% below the expected prevalence of 7.3% reported by the CDC for the US Black and/or African American population. Twenty-one of 162 (13%) Black athletes left the question blank, indicating either lack of knowledge of SCT status or declined to answer (Table 2). There were no additional SCT “yes” responses among the 239 non-Black athletes, although there were an additional 19 blank responses, bringing the overall “blank” responses to 11%.

**Table 2:** Question 40: Have you ever been diagnosed with sickle cell trait? *Expected prevalence per CDC [4].

Race (#)	No	Yes	Blank	Reported Prevalence	Expected Prevalence*
Asian (7)	7	0	0		
Biracial (2)	2	0	0		
Black (162)	135	6	21	3.7%	7.3%
Hispanic (5)	4	0	1		
Other (13)	10	0	3		
Unknown (97)	88	0	9		
White (115)	105	0	10		
Total (401)	351	6	44	1.5%	1.5%

Further analysis into blank non-responses compared differences between the sickle cell responses to the 10 AHA cardiac screening questions (Table 3). There were a total of 49 blank responses to questions 39 and 40 regarding SC status, five for SC anemia, and 44 for SC trait. A similar percentage of blanks was consistent across races at ~10% for SCT. In contrast, there were only 18 total blank responses to all combined 10 AHA cardiac screening questions.

**Table 3:** Summary of all blank responses AHA, American Heart Association.

Race (#)	AHA Q9-18	Q39	Q40
Asian (7)	0	0	0
Biracial (2)	0	0	0
Black (162)	5 (3.1%)	3 (1.9%)	21 (13.0%)
Hispanic (5)	0	0	1 (20.0%)
Other (13)	0	0	3 (23.1%)
Unknown (97)	5 (5.2%)	2 (2.1%)	9 (9.3%)
White (115)	10 (8.7%)	0	10 (8.7%)
Total (401)	18 (4.5%)	5 (1.2%)	44 (11.0%)

## Discussion

SCT is known to have caused death and disability in athletes in both high school and collegiate settings. The value of SCT identification and targeted training modifications has not fully been proven across all the athlete ages and sports, but mandatory screening in NCAA Division I football athletes has been shown to decrease mortality from exertion-related sickling, while strict attention to heat and hydration has been demonstrated to prevent death in US military recruits [7,10]. It is reasonable to suggest a combination of proper screening, exertional precautions, and prompt recognition, and the treatment of early signs or symptoms gives an athlete the best chance to avoid morbidity and mortality associated with SCT.

If identification of athletes with SCT is effective in preventing mortality, then a proper screening of high-risk groups, including high-school athletes, must be considered as a part of the preparticipation screening. According to the National Center for Catastrophic Sport Injury Research data, there have been seven deaths related to exertional sickling in middle-/high-school athletes in the last 10 years (2008-2017) [12]. This study highlights these at-risk youth athletes who are not being properly screened in PPEs, potentially placing them in situations that could cause serious illness or death related to unknown SCT status.

If a governing body determines sickle cell screening to be unethical or discriminatory, then universal precautions should be enforced at all levels, and all screening methods should be abandoned. As long as there are high-school athletes exerting themselves in different climates, conditioning levels, altitudes, and various access to healthcare professionals, this method will incredibly be difficult to monitor/enforce and will continue to place athletes with SCT at risk. One has to consider replacing current inadequate screening methods, such as the questionnaire format inconsistently utilized in the state of Florida, with improved options to clearly define SCT status. If blood testing is not available, the authors suggest including a question to assess if newborn SCT screening status is known and possibly adding consent to obtain state medical records if not. Further counseling should be offered if unknown or unsure. 

Within the next few years, the Florida Department of Health will have all high-school athletes’ newborn SCT screening results available in an accessible database. Athletes born since 2006 can have their status requested with no additional cost or blood draw. If successful, these recommendations could be adopted by other states. It is the responsibility of each healthcare professional involved in PPEs to educate athletes and their families about the risks of SCT in sports and to work with local and state authorities to ensure proper screening of SCT along with appropriate precautions to help prevent any future catastrophic events in young athletes. 

Limitations

Electronic medical records were utilized to review demographics and obtain race status; therefore, the race was not determined for any athlete not previously registered in the UF Health network (n = 97). Although, if even a portion of the respondents for which race could not be confirmed are Black respondents, the SCT prevalence could be even lower than the reported 3.7% (anywhere from 2.3% to 3.7%) as no additional unknown race athletes responded “yes” to SCT status. 

Additionally, there is a difference in language between the categories of “Black” race captured in our data versus the “Black or African American” language describing the data captured by the CDC. The CDC also did not offer any biracial category. These inconsistencies may have affected the categorical percentages represented as screening positive for SCT in the CDC sample and, therefore, altered the outcome of comparisons to our results. 

There is also a concern of possible repeat athletes participating from year to year since the athletes’ records were de-identified following each PPE session. This could potentially falsely increase the study numbers and distort the interpretation of data. 

There were a high number of blank responses to SCT status (n = 44). It is unknown if the athletes declined to answer due to potential discrimination or lack of knowledge of their SCT status.

## Conclusions

This appears to be the first study evaluating the efficacy of self-report SCT screening in a youth population. Prevalence rates by self-report are lower than accepted statistics in this selected population. Response rates are inconsistently answered and are lower than other cardiac screening response rates. Reasons for this are unclear but may include SCT status unknown to parents or athletes, or false reporting due to possible discrimination. These findings highlight the necessity for improved screening methods for SCT in the high-school athlete population. Further research is needed into alternate screening tools and their effect on the recognition and treatment of SCT conditions. 
